# Cognitive Decline and Financial Well-Being: Evidence From the Cognitive Economics Study

**DOI:** 10.1093/geroni/igaa057.1536

**Published:** 2020-12-16

**Authors:** Yuci Zhou

**Affiliations:** Massachusetts Institute of Technology, Cambridge, Massachusetts, United States

## Abstract

Maintaining financial well-being is important for older adults, as they are generally decumulating wealth at this stage of life. We use survey data from the Cognitive Economics Study (CogEcon, 2008-2017) and Cognition and Aging in the USA Study (CogUSA, 2007-2014) to investigate the impact of cognitive decline on financial well-being of older adults in the United States. In our sample (N = 976), with an average starting age of 65 years old, we use linear regression, then add results including a control function approach to correct our estimates for sample selection and attrition. In particular, those who have poor health outcomes (including death) disproportionately attrit from the study and may bias the results of a naïve regression. The naïve regression results yield no relationship between cognition and financial well-being; however, after controlling for attrition, we find that a decline in crystallized intelligence is associated with a decrease in financial well-being, and changes in fluid intelligence are not associated with changes in financial well-being.

